# Engineering exosomes for cancer therapy - Modification technologies and subcellular targeting strategies: A review

**DOI:** 10.1016/j.ijpx.2025.100473

**Published:** 2025-12-31

**Authors:** Fengbo Liu, Chengxing Xia, Hang Yu, Xiaofang Yang, Liping Ge, Chunwei Ye

**Affiliations:** Department of Urology, The Second Affiliated Hospital of Kunming Medical University, Kunming, China

**Keywords:** Exosome, Cancer, Therapy, Modification, Subcellular targeting

## Abstract

Exosomes are secreted lipid bilayer vesicles of 30–150 nm in diameter. Their low immunogenicity, excellent biocompatibility, and inherent targeting capability make them a promising drug delivery vehicle for cancer therapeutics. However, the use of natural exosomes is still challenging for therapeutic applications, including limited targeting precision and drug-loading efficiency, necessitating engineered modification strategies to optimize their performance. To further enhance exosome targeting capacity, recent studies have explored precision delivery strategies targeting subcellular structures such as lysosomes, nuclei, mitochondria, and the endoplasmic reticulum, thereby improving exosome therapeutic potential. This review systematically summarizes the core advantages of exosomes as drug carriers, elaborates on their engineering modification methods, and highlights recent advances in strategies to improve exosomes targeting of subcellular structures to enhance antitumor efficacy. The review aims to provide a theoretical foundation and technical guidance for developing exosome-based precision therapies for cancer.

## Highlights

• A comparison between exosomes and other nanomaterials.
• Engineering modifications of exosomes as anti-tumor drug carriers: classifications and examples.
• Targeting subcellular structures of tumors may represent a direction for further enhancing anti-tumor efficacy.
• Methods and examples for targeting various subcellular units.

## Graphical abstract

**Figure:**
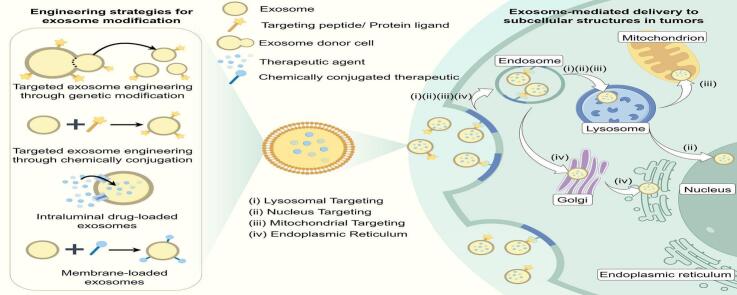

## 1 Introduction

Exosomes are 30–150 nm lipid-bilayer extracellular vesicles, endogenously secreted by nearly all cell types. They are ubiquitously present in biological fluids including blood, saliva, and urine. These vesicles mediate intercellular communication by transporting bioactive molecules, including proteins, lipids, DNA, mRNAs, and microRNAs, from parent cells (Johnsen et al., 2014; Kalluri and Lebleu, 2020). In the field of oncology, exosomes have gained significant attention due to their exceptional drug-loading capacity and modifiable properties (Bray et al., 2024). They serve as natural carriers to deliver chemotherapeutic agents (Chen and Lan, 2024; Watson et al., 2015), immunotherapeutics (Nassief et al., 2024; Mukherjee et al., 2021; Li et al., 2024), and targeted drugs to tumor cells (Li et al., 2022b; Park et al., 2016; Harada et al., 2023; Modi et al., 2022). Engineered as multifunctional nanoplatforms, exosomes now offer novel approaches for targeted drug delivery and immunomodulation in cancer therapy.

Exosomes exhibit unique advantages over other nanocarriers. As endogenous nanovesicles, exosomes have natural low immunogenicity and excellent biocompatibility, significantly outperforming synthetic nanomaterials. Their size and specialized membrane structure confer exceptional tissue-penetrating ability, enabling the exosomes to efficiently cross critical physiological barriers such as the blood-brain barrier (Cheng and Hill, 2022; Wang et al., 2021c; Li et al., 2022a). More importantly, exosomes carry donor cell-specific membrane proteins (e.g., CD47, integrins) on their surface, providing inherent targeting properties (Guo et al., 2024a). These characteristics of targeting capability, penetration efficiency, and biosafety make exosomes ideal drug carriers with great potential for tumor-targeted therapy.

However, exosomes are typically designed to target whole tumor cells, leading to nonspecific drug distribution around or within the cytoplasm, resulting in drug loss. Currently, strategies to improve the delivery efficiency of exosomes primarily focus on optimizing targeting mechanisms or decreasing clearance during systemic circulation. Among the different strategies, exosomes targeting subcellular structures have emerged as a promising precision delivery strategy with significant potential (Ye et al., 2025). Unlike conventional approaches, subcellular targeting enhances existing targeting mechanisms while extending the functional pathway of exosomes after cellular internalization, thus improving intracellular trafficking of both exosomes and their cargo (Cheng et al., 2025). Tools, such as target-directed peptides, aim to enhance the efficacy of drugs within cells, addressing the current limitations in secondary intracellular targeting exhibited by most exosome-based drug delivery systems (Yang et al., 2022). Taken together, subcellular targeting and its associated tools provide new insights for intracellular targeting and precision therapy research. Given the dearth of studies on subcellular targeting for exosome-based drug delivery and the lack of comprehensive reviews in this area, here we systematically describe the potential and advantages of subcellular targeting, thereby opening new avenues for the application of exosomes in tumor therapy.

In summary, exosomes have unique drug delivery advantages and show promising therapeutic potential in targeting subcellular structures. This review aims to establish a comprehensive exosome modification-targeting-efficacy correlation map by systematically examining the biological characteristics of exosomes, engineering strategies for exosome-based drug carriers, and recent advances in subcellular targeting approaches. By summarizing current research and future perspectives, we aim to provide new insights into the development of more precise subcellular-targeting exosome therapies and offer theoretical support to advance exosomes as drug-delivery vehicles from basic research to clinical applications in tumor precision medicine.

## 2 Biological characteristics and engineering strategies of exosomes

### 2.1 Biological characteristics of exosomes

Exosomes demonstrate significant advantages over conventional synthetic delivery systems such as liposomes, polymeric nanoparticles, and inorganic nanoparticles. These advantages include excellent biocompatibility, inherent targeting capabilities, and drug-loading capacity. Exosomes naturally express signaling proteins such as PD-1 (Programmed cell death protein 1) on their surface, which confer intrinsic active targeting properties (Pan et al., 2020; Sarkar et al., 2024; Zhou et al., 2021). In contrast, synthetic materials require additional modifications (e.g., the addition of targeting ligands such as the Arginine-Glycine-Aspartate (RGD) peptide), which may compromise carrier stability. In terms of drug-loading strategies, exosomes can protect drugs by encapsulating them, while also undertaking membrane modifications for multi-functionalization. In comparison, inorganic carriers, such as gold nanorods, have limited loading options and are prone to drug leakage. Exosomes efficiently cross biological barriers to facilitate tissue penetration. For example, exosome-like particles isolated from ginseng can cross the blood-brain barrier and specifically target gliomas (Kim et al., 2023). On the flip side, inorganic nanoparticles (e.g., silica) are more susceptible to clearance by mononuclear phagocytes. Exosomes, as endogenous vesicles, exhibit minimal immunogenicity and high biocompatibility (Record et al., 2018). In contrast, liposomes may trigger complement activation, and polymeric like poly (lactic-*co*-glycolic acid) (PLGA) nanoparticles introduce risks such as byproducts of acidic degradation. From a safety perspective, clinical data indicate that exosome-based delivery systems have significantly lower adverse reaction rates compared to cationic liposomes (Khidr et al., 2025; Mondal et al., 2023). Of note, exosomes may inherit membrane properties from their parent cells. For example, dendritic cell-derived exosomes significantly inhibited tumor growth through immune pathways—a biological activity that synthetic carriers cannot replicate (Zitvogel et al., 1998; Escudier et al., 2005). And Eexosomes themselves possess regulatory capabilities. For instance, exosomes derived from M2-type macrophages can enhance flap survival by promoting angiogenesis(Luo et al., 2024). Moreover, exosomes exhibit unique biodistribution characteristics, with a longer circulation half-life than synthetic alternatives (Moholkar et al., 2023). Taken together, these advantages establish exosomes as a representative next-generation intelligent delivery system (Table 1).

**Table 1:** Comparison of biological characteristics between exosomes and other nanomaterials.

Examples	Targeting ability	Drug-loading capacity	Penetration ability	Biocompatibility	Safety profile	Circulation half-life	Additional notes	References
Activated T cell-derived exosomes, HEK293T cell-derived exosomes	Natural targeting, ligand-receptor targeting, peptide-mediated targeting	High loading capacity (strong protective capability)	High	High	High	Long (immune evasion)	Retention of parental cell function	(Qiu et al., 2021; Kamerkar et al., 2022)
Liposomes	Passive targeting (EPR effect)	High loading capacity	Moderate (Size/PEGylation-dependent)	High (phospholipids)	Moderate (lipid oxidation products)	Moderate	Well-established fabrication	(Liu et al., 2022; Garanina et al., 2023; Zhan and Wang, 2018)
PLGA nanoparticles	Passive targeting	High loading capacity (material-dependent)	Moderate, EPR-dependent	Moderate (acidic degradation products of PLGA)	Moderate (polymer degradation products)	Short	Superior sustained-release properties	(Zhang et al., 2020b; Xiao et al., 2022)
MSNs, AuNRs	Passive targeting	High loading capacity (limited loading types)	Low (reticuloendothelial system clearance)	Low (risk of metal ion accumulation)	Low (risk of prolonged retention)	Short (rapid hepatic uptake)	Ease of modification	(Zhao et al., 2024; Huang et al., 2021)

Abbreviations list: PLGA, Poly(lactic-*co*-glycolic acid); MSNS, Mesoporous silica nanoparticles; AuNRs, Gold nanorods; EPR, Enhanced Permeability and Retention.

### 2.2 Exosome engineering strategies

The exosome natural characteristics underpin the potential of exosomes for tumor targeting and drug delivery. However, natural exosomes face limitations in quality control: even when derived from the same cell type, variations exist in their size, protein expression, and cargo composition (e.g., targeting proteins). Typically, natural exosomes exhibit low expression levels of specific targeting proteins and carry a variety of non-targeting proteins, which may lead to off-target effects or immune clearance (Sahoo et al., 2024). Moreover, their drug-loading efficiency is relatively low, especially for hydrophilic drugs (Huang et al., 2023). For instance, (Hosseini et al., 2022) achieved only about 13 % DOX loading efficiency through co-incubation, whereas (Ebrahimian et al., 2022) employed an engineered strategy (combining incubation, surfactant treatment, and freeze-thaw cycles) to increase thymoquinone loading efficiency to 60 %, highlighting both the limitations and the potential for improvement of natural exosomes. In summary, as unmodified exosomes are insufficient to meet tumor therapeutic needs, exosomes should be engineered to enhance their performance. Current exosome modification strategies available to rationally design exosomes mainly focus on developing precise targeting modification or multifunctional modification strategies. These studies have laid an important foundation to construct next-generation exosome-based tumor treatment systems. In the following sections, we review and summarize the main technical methods for targeted modification and functionalization of exosomes.

Engineering exosomes to achieve precise targeting addresses key limitations of their natural counterparts in tumor therapy, such as low targeting efficiency and source dependency. To date, various engineering strategies have been developed,which can be broadly categorized by the target of engineering, engineering strategies mainly fall into two classes: internal engineering via genetic manipulation of donor cells and external engineering via direct modification of isolated exosomes (Table 2) (Uddin et al., 2023; Zhou et al., 2022; Liang et al., 2024).

**Table 2:** Exosome engineering strategies for targeted applications.

Source of exosomes	Tumor types	Targeting strategy	Modification methods for targeted materials	References
Mouse macrophage cells J774A.1	Breast cancer, osteosarcoma	Ligand-receptor binding (naturally targeted)	Unmodified	(Rayamajhi et al., 2019)
BM-MSC	PDAC	Ligand-receptor binding (naturally targeted)	Unmodified	(Zhou et al., 2021)
Ginseng	Breast cancer	Ligand-receptor binding (naturally targeted)	Unmodified	(Gu et al., 2024)
MEFs, BMDCs	Glioblastoma	Ligand-receptor binding, electrostatic force (CDX peptide, CREKA peptide)	Genetic engineering (permeabilization, transfection)	(Yang et al., 2020)
HEK293 cells	Colon cancer, hepatocellular carcinoma	Ligand-receptor binding (PTGFRN gene)	Genetic engineering (electroporation, transfection)	(Kamerkar et al., 2022)
HEK293T cells	Her2-positive gastric cancer	Ligand-receptor binding (HER2 affibody)	Genetic engineering (lentiviral transduction)	(Liang et al., 2020)
HEK293T cells	Hepatocellular carcinoma	Ligand-receptor binding (CD47)	Genetic engineering (chemical transfection)	(Du et al., 2021)
CT26 cells	Colon cancer	Ligand-receptor binding (CD47)	Genetic engineering (lentiviral transduction)	(Cheng et al., 2021)
U937 monoblastic cells	Prostate cancer	Ligand-receptor binding (anti-PSMA peptide)	Genetic engineering (nucleofection, lentiviral transduction)	(Severic et al., 2021)
HEK-293 T cells	ATC	Ligand-receptor binding (tumor-penetrating peptide iRGD)	Genetic engineering (lentiviral transduction)	(Wang et al., 2022)
Dendritic cells (DC)	Hepatocellular carcinoma	Ligand-receptor binding (P47-derived peptide)	Biospecific recognition (CP05-CD63)	(Zuo et al., 2022)
HEK293T cells	B-cell lymphoma	Ligand-receptor binding (PEG, CP05 peptide)	Chemical cross-linking (maleimide-thiol coupling)	(Wan et al., 2022)
HEK293T cells	KRAS-mutated colorectal cancer	Ligand-receptor binding (CTX)	Lipid insertion (DSPE-PEG-NHS)	(Xiang et al., 2025)
Milk	Colon Cancer, lung Cancer, cervical Cancer	Ligand-receptor binding (folate)	Lipid insertion (DSPE-PEG2000-folate)	(Liang et al., 2024)
THP-1 macrophages	cervical cancer	Ligand-receptor binding (RGD peptide, folate)	Lipid insertion, chemical cross-linking (DSPE-PEG-RGD, DSPE-PEG-SH, Au—S bond formation, NHS–amine coupling)	(Wang et al., 2018)
THP-1 macrophages	Cervical cancer, breast cancer	Magnetic force (Fe_3_O_4_)	Lipid insertion (DSPE-PEG-Biotin)b	(Wang et al., 2021b)
Human placental mesenchymal stem cells (MSCs)	Breast cancer	Magnetic force (carboxylated Fe₃O₄)	Chemical cross-linking, physical connection (carboxyl-amino coupling, Fe₃O₄)	(Xu et al., 2024)
C6 rat glioma cell	GBM	Ligand-receptor binding (T7 peptide)	Membrane hybridization (PHR, EM)	(Lee et al., 2025)
M1 macrophages	Cutaneous melanoma	Ligand-receptor binding (naturally targeted)	Membrane hybridization (liposome, exosome)	(Zhou et al., 2025)
Raw264.7	Malignant glioma	Ligand-receptor binding (NRP-1, RGE peptide)	Chemical cross-linking, click chemistry (EDC/NHS, click)	(Jia et al., 2018)

Abbreviations list: BM-MSC, Bone marrow mesenchymal stem cells; PDAC, Pancreatic ductal adenocarcinoma; MEFs, Mouse embryonic fibroblasts; BMDCs, Bone marrow-derived dendritic cells; CDX peptide, Caudal-type homeobox transcription factor-derived peptide; PTGFRN, Prostaglandin F2 receptor negative regulator gene; HER2 affibody, Human epidermal growth factor receptor 2-binding affibody molecule; LAMP2, Lysosome-associated membrane protein 2; GFP, Green fluorescent protein; PSMA, Prostate-specific membrane antigen; ATC, Anaplastic thyroid carcinoma; PEG Polyethylene glycol; CTX, Cetuximab; RGD, Arginine-Glycine-Aspartic acid; PDA, Polydopamine; GBM Glioblastoma; PHR, Polyamidoamine dendrimer conjugated with Histidine and aRginine; EM, Exosome-membrane; Click, Copper-catalyzed Azide-Alkyne Cycloaddition.

Genetic engineering involves genetic modification at the donor cell level (e.g., lentiviral transfection or electroporation nucleofection) to produce exosomes carrying specific proteins (Wang et al., 2020; Nam et al., 2020). For example, lentiviral transfection was used to overexpress CD47 in CT26 colon carcinoma cells, generating exosomes with prolonged circulation time and enhancing tumor targeting through the CD47-SIRPα pathway (Cheng et al., 2021). A different study compared electroporation with lentiviral transfection efficiency and reported greater efficiency with electroporation while transfection enabled stable long-term expression in U937 macrophage cells (Severic et al., 2021). The overall advantages of this method lie in its ability to preserve the structural integrity and biological functions of exosomes while achieving relatively stable and uniform modifications. However, it is technically complex and time-consuming. Additionally, varying transfection efficiencies across different cell lines often lead to low exosome yields or inconsistent modification efficiency. Moreover, this approach typically relies on protein overexpression, which may suffer from unstable expression efficiency(Huang et al., 2023). On the other hand, surface engineering at the exosome level — involving modifications with ligands, peptides, or aptamers — can be further subdivided into more specific approaches, including click chemistry (bioorthogonal reactions), chemical cross-linking (chemical bonding), lipid insertion (hydrophobic interaction), electrostatic adsorption (charge-based), and membrane hybridization (fusion with biomembranes), among others.Chemical crosslinking utilizes chemical reactions (such as amine-carboxyl condensation) to connect exosomes with ligands, resulting in strong bonding but poor reaction specificity. Lipid insertion involves the insertion of ligands modified with hydrophobic tails (such as DSPE or cholesterol) into the exosomal membrane, which is simple to operate but leads to weaker binding affinity. (Wang et al., 2018) employs a donor cell-assisted membrane modification strategy to engineer multifunctional exosomes. Macrophage donor cells are first incubated with DSPE-PEG-RGD and DSPE-PEG-SH, which integrate into the cell membrane via insertion, exposing RGD peptides for active tumor targeting and sulfhydryl (-SH) groups as conjugation anchors. The secreted exosomes inherit these modifications, yielding pre-functionalized RGD-Exos-SH. The surface-exposed SH groups then allow covalent conjugation of gold nanorods (AuNRs) via Au—S bonds for photothermal therapy. Folic acid (FA) is further attached to the AuNR surface through NHS-amine coupling, establishing a dual-ligand (RGD/FA) targeting system. Finally, doxorubicin (DOX) is loaded into the exosomes via electroporation, resulting in the complete therapeutic nanocomplex. And the membrane hybridization strategy based on extrusion involves rupturing and reassembling membranes, enabling the integration of membrane features from different sources. However, exosomal membranes subjected to fragmentation may experience functional loss (Hu et al., 2021). For instance, (Pei et al., 2024) employed membrane extrusion technology to fuse platelet membrane fragments, obtained through repeated freeze-thaw cycles and ultrasonication, with extracellular vesicles derived from M2 macrophages. This strategy leverages the homing ability of platelet membranes to target vascular injury and inflammatory sites, while utilizing M2 macrophage-derived vesicles to deliver nucleic acids and other payloads to regulate immunity and suppress inflammation, thereby synergistically treating viral myocarditis. Click chemistry utilizes highly reactive functional groups that undergo cycloaddition reactions to form stable covalent bonds. This approach offers high specificity but may lack universality in its applicability. (Xu et al., 2020) first reacted DBCO-NHS with the amino groups on the surface proteins of exosomes to anchor the click chemistry group DBCO onto the membrane. Subsequently, an azide-modified fluorescent dye was added, and stable labeling was achieved through the efficient cycloaddition reaction between DBCO and azide. Other methods involve loading materials based on physical properties such as electrical charge. For example, carboxylated Fe₃O₄ nanoparticles (NPs) combined with exosomes enabled magnetic-guided targeted delivery through electrostatic adsorption (Xu et al., 2024). However, these approaches are highly susceptible to interference from surrounding charges and other environmental factors, leading to off-target effects.

In summary, the selection of optimal strategies or combinations must be based on therapeutic requirements to balance efficiency and safety.

Beyond targeting, functionalizing exosomes by loading therapeutic cargo is an equally critical objective. Exosomes demonstrate strong capacity to deliver diverse therapeutic agents to tumor cells, including chemotherapeutic drugs (Liu et al., 2023; Guo et al., 2024b), dynamic therapy components (Gu et al., 2024; Du et al., 2021), photothermal therapy materials (Han et al., 2025), and immunotherapeutic agents (Li et al., 2023a; Huang et al., 2022; Phung et al., 2020). However, effective loading of these drugs requires specific modifications of the exosomes. Based on differences in drug physicochemical properties and required spatiotemporal action, modification methods can be classified into lumen loading and membrane loading depending on the location (Table 3).

**Table 3:** Exosome engineering for drug loading.

Source of exosomes	Tumor types in research	Treatment modality	Modification methods		References
Mouse macrophage cell J774A.1	Breast cancer, osteosarcoma	Chemotherapy (DOX)	Co-incubation		(Rayamajhi et al., 2019)
HEK293 cells	Colon cancer, hepatocellular carcinoma	Immunotherapy (STAT6 ASO)	Co-incubation		(Kamerkar et al., 2022)
HEK293Tcells	Hepatocellular carcinoma	Chemotherapy (Erastin), photodynamic therapy (Rose Bengal)	Sonication		(Du et al., 2021)
MSCs	Breast cancer	Chemotherapy (DOX)	Sonication		(Xu et al., 2024)
Ginseng	Breast cancer	PDT (ICG)	Sonication		(Gu et al., 2024)
BMSCs	Breast cancer	Immunotherapy (LJP)	Sonication		(Zhang et al., 2025)
HEK293T cells	5-FU-resistant colon cancer	Chemotherapy (5-FU), Gene therapy (miR-21i)	Electroporation		(Liang et al., 2020)
HEK293Tcells	B-cell lymphoma	PDT (RB), Chemotherapy (DOX)	Electroporation		(Wan et al., 2022)
BM-MSC	PDAC	Immunotherapy (Galectin-9 siRNA, OXA prodrug)	Electroporation, chemical conjugation		(Zhou et al., 2021)
HEK-293 T cells	ATC	Chemotherapy (DOX), internal irradiation therapy (^131^I)	Chemical conjugation (chloramine-T method)		(Wang et al., 2022)
Dendritic cells	Hepatocellular carcinoma	Immunotherapy (AFP212-A2, AFP N1ND-N)	Chemical conjugation		(Zuo et al., 2022)

Abbreviations list: Dox, Doxorubicin; ASO, antisense oligonucleotide; RB, Rose Bengal; ICG, Indocyanine Green; LJP, *Lonicera japonica* polysaccharide; 5-FU, 5-Fluorouracil; PDT, Photodynamic therapy; OXA, Oxaliplatin; AFP, Alpha-Fetoprotein.

Commonly used methods include co-incubation, sonication, membrane modification, extrusion, electroporation, and saponin permeabilization. Among these, co-incubation is generally suitable for lipophilic drugs. Encapsulation of Doxorubicin (DOX) into exosome-mimetic nanovesicles was successfully achieved by co-incubation (Rayamajhi et al., 2019). Sonication utilizes cavitation effects to create transient and repairable pores in the exosomal membrane. This method can also be applied to hydrophilic drugs with relatively high efficiency, but it may cause damage to the exosomal membrane (Kumar et al., 2023). (Zhang et al., 2025) achieved higher loading efficiency by incorporating *Laminaria japonica* polysaccharides into BMSC-derived exosomes through sonication. However, membrane modification strategies (analogous to the surface engineering described above for targeting) are employed for components requiring early-phase activity or exhibiting high stability. For example, exosome surface tyrosine residues were labeled with ^131^I using the chloramine-T method, enabling targeted radiotherapy (Wang et al., 2022). Electroporation generates hydrophilic pores, which are particularly suitable for nucleic acid-based drugs, but may also lead to aggregation of charged macromolecules (Wan et al., 2022). Saponin permeabilization works by specifically binding to cholesterol in the membrane to form reversible pore complexes. This method operates under mild conditions but carries safety risks such as hemolysis (Haney et al., 2015). It should be noted that the aforementioned methods present challenges in terms of process stability or safety when considered for industrial-scale applications. The discussion here is limited to laboratory-scale preparation.

## 3 Exosome-based strategies and mechanisms for targeting tumor subcellular structures

The engineering modifications described above have significantly enhanced the targeting specificity and therapeutic efficacy of exosome-based delivery systems. However, the inherent biological complexity of tumors necessitates more sophisticated drug delivery approaches to achieve optimal therapeutic outcomes. Current research approaches predominantly focus on utilizing exosomes to target tumor cells as an undifferentiated population, followed by therapeutic application of the engineered vesicles. Exosomes can enter tumor cells through multiple distinct pathways. The most well-characterized route is the classical endosomal-lysosomal pathway, while alternative mechanisms include the endosomal-Golgi-endoplasmic reticulum route and direct plasma membrane penetration (Arya et al., 2024).

An inherent limitation of current exosome-mediated delivery systems is significant drug wastage. This inefficiency primarily stems from two key factors. First, after tumor cell targeting is achieved, the lack of subsequent subcellular targeting modifications results in nonspecific distribution of exosomes either around tumor cells or within cellular compartments. Second, different exosome complexes may be internalized through distinct pathways, necessitating selective modifications tailored to their specific internalization mechanisms to enhance delivery efficiency. Furthermore, even following successful cellular internalization, exosomes are frequently subjected to clearance or random intracellular distribution due to inadequate targeting specificity. This limitation reduces local drug concentrations, contributing to drug wastage and potentially inducing drug resistance or other adverse effects. To overcome these bottlenecks, recent studies have focused on developing secondary targeting strategies directed toward tumor cell subcellular structures (Liang et al., 2024). These approaches aim to enhance directional drug delivery efficiency and consequently increase local drug concentrations at target sites (Liu et al., 2024; Wang et al., 2021a; Zhang et al., 2020a).

Overall, subcellular targeting holds greater advantages over passive targeting and cellular targeting (Table 4**)**. Passive targeting suffers from low precision, which can easily damage normal tissues and results in insufficient drug concentration at tumor sites. Cellular targeting enhances targeting efficiency through modifications and serves as the foundation for subcellular targeting. As an extension of cellular targeting, subcellular targeting enables secondary localization after cellular engagement, allowing for precise drug delivery to specific organelles, thereby improving therapeutic efficacy and preventing drug degradation(Li et al., 2023b; Liu et al., 2020).

**Table 4:** Comparison of targeted strategies in exosome-based cancer therapy.

Type	Targeting dimension	Targeting mechanism	Precision	Representative targeting molecules/ligands	References
Passive targeting	Tumor tissue	Enhanced permeability and retention (EPR) effect; prolonged circulation	Low	mPEG2000, CD47	(Wan et al., 2022)
Cellular targeting	Specific tumor cells	Ligand-receptor interaction, antigen-antibody recognition	Medium	Anti-PSMA peptide (prostate cancer), anti-HER2 antibody (breast cancer, colon cancer)	(Severic et al., 2021), (Liang et al., 2020)
Subcellular targeting	Specific organelles	Signal peptide-mediated transport, organelle-specific ligands, physical/chemical triggers	High	TPP peptide, NLS peptide	(Cheng et al., 2019), (Nguyen Cao et al., 2023)

As a subcellular targeting vehicle, exosomes exhibit significant advantages over synthetic nanomaterials: they can protect nucleic acid-based drugs from enzymatic degradation and, owing to their inherent biocompatibility and membrane-crossing ability, reduce delivery losses. In terms of targeting modification, conventional nanomaterials rely on relatively singular approaches, limited by coupling efficiency and ligand selection(Liu et al., 2024). In contrast, exosomes can be engineered through multiple strategies such as membrane modification, genetic engineering, and click chemistry, offering a broader range of ligand choices. Their favorable biocompatibility also helps preserve the native conformation and activity of ligands, thereby enhancing targeting precision and therapeutic efficacy(Li et al., 2023b).

The following section systematically reviews exosome vectors for subcellular targeting (lysosomes, nuclei, mitochondria, and endoplasmic reticulum) in cancer therapy and the approaches taken as guided by the unique biological properties of each subcellular structure.

### 3.1 Lysosomes

**Fig. 1:**
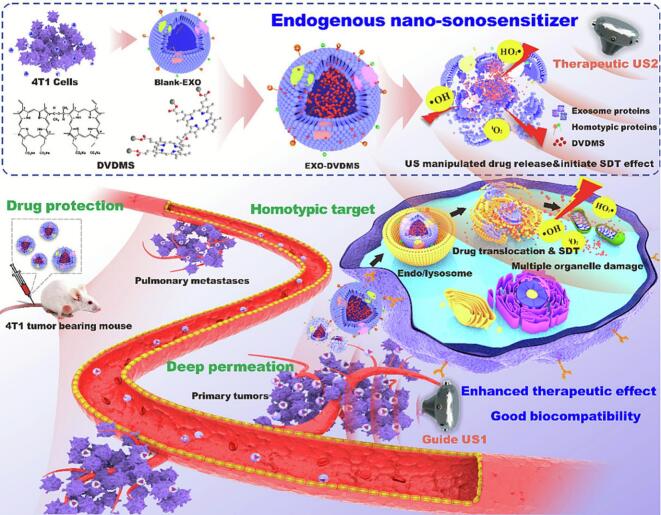
Ultrasound-triggered EXO-DVDMS selectively targets tumors, disrupts lysosomes, and releases DVDMS into the cytoplasm. The DVDMS boosts the production of reactive oxygen species (ROS) and enhances sonodynamic therapy (SDT) efficacy. Reprinted from (Liu et al., 2019); licensed under CC BY 4.0.

Lysosomes represent the primary destination for exosomes following cellular internalization **(**Fig. 1**)** and the lysosomes-exosomes interaction mechanisms provide critical insights for tumor-targeted therapy. Lysosomes recognize exosome surface markers (e.g., CD63, CD9) as degradation substrates and capture exosomes through the endosomal-lysosomal pathway (Li et al., 2020). This presents both challenges and opportunities for therapeutic applications. The main challenge lies in the potential degradation of exosomes and their cargo by lysosomal enzymes, which will significantly compromise therapeutic efficacy. However, this limitation can be overcome by rational engineering to develop smart drug release systems. One successful demonstration of this strategy is the ultrasound-responsive nanosonosensitizer (Exosome-Loaded Sinoporphyrin Sodium, EXO-DVDMS) which was developed through innovative utilization of lysosomal physiological characteristics (Liu et al., 2019). Initially, homologous tumor cell-derived exosomes were used to ensure targeted delivery. Then, exosomes were transported to lysosomes via endocytosis. Subsequently, the acidic lysosomal environment (pH 4.5–5.0) triggered a pH-sensitive mechanism. Sodium carboxylate groups (-COONa) were protonated to hydrophobic -COOH, resulting in exosome membrane destabilization and DVDMS release. Finally, ultrasound cavitation effects were applied to further disrupt lysosomal membranes, which promoted DVDMS escape into the cytoplasm (Liu et al., 2019). This multistage strategy reduced lysosomal clearance of DVDMS while enabling synergistic tumor killing through combined DVDMS activity and lysosomal enzyme release. Furthermore, extended in vivo-derived data (the BALB/c mouse subcutaneous 4 T1 breast cancer xenograft model) revealed a 70 % reduction in tumor mass in an ultrasound-treated group (U2, which induced lysosomal membrane disruption) compared to a non-U2 group. The universal applicability of this strategy was confirmed with the development of a magnetic-targeting exosome system that enabled doxorubicin (DOX) release. (Xu et al., 2024). Exosomes loaded with DOX, coupled with drug release in an acidic environment, eliminated DOX-induced body weight loss and reduced the tumor weight to 20 % of that for the group treated with DOX alone(the orthotopic MDA-MB-231 human breast cancer xenograft model). Lysosomes were utilized as both the final destination for exosome internalization and the acidic trigger for drug release. Taken together, two key principles are demonstrated by these studies: (1) exosomes are naturally predisposed to lysosomal targeting through their intrinsic internalization pathways, and (2) the acidic lysosomal environment can be effectively utilized as an ideal trigger for controlled drug release (Fig. 1).

In contrast to the aforementioned strategy of utilizing the lysosomal microenvironment for drug release, a recent study proposed an alternative innovative approach, i.e., enhancing exosome-based drug delivery by blocking the endosome-lysosome pathway. (Ashraf et al., 2022) used exosomes derived from normal mammary epithelial cells (MCF10A) or osteoblasts (hFOB 1.19) as carriers to encapsulate silver‑copper nanoparticles (AgCu-NPs), which were then conjugated with wheat germ agglutinin (WGA). This system leverages the homologous targeting capability of exosomes for precise delivery, while the fusion of late endosomes with lysosomes is effectively inhibited through WGA-mediated regulation of Rab7a protein and deubiquitinase coordination. Consequently, AgCu-NPs are prevented from entering lysosomes, thereby avoiding oxidative inactivation caused by the acidic environment. Additionally, the metallic AgCu-NPs released via multivesicular body (MVBs)-plasma membrane fusion, catalyzed the generation of reactive oxygen species (ROS). Simultaneously, Cu^2+^-induced lysosomal membrane disruption led to the release of hydrolytic enzymes, resulting in combined cytotoxic effects, including DNA damage, mitochondrial dysfunction, and apoptosis. This study demonstrated that modulation of the endosome-lysosome pathway significantly enhanced the therapeutic efficacy of exosome-based delivery systems (Ashraf et al., 2022). From in vitro experimental data (MCF10A cells and osteoblast cells hFOB 1.19), cells treated with WGA-conjugated exosomes exhibited a decrease in both Pearson's correlation coefficient and Mander's coefficient from approximately 0.8 to ∼0.1. This supports the robust ability of conjugated exosomes to evade lysosomes. Concomitantly, the tumor cell survival rate decreased to approximately 75 %. Similarly, (Zhang et al., 2024) utilizes SR-B1 (Scavenger Receptor Class B Type I) high density lipoprotein receptor-mediated endocytosis, enabling exosomes to bypass lysosomal degradation and undergo Golgi-directed transport to the ER. Such targeted delivery significantly enhanced siRNA gene silencing efficiency to 96.78 % at the mRNA level and 94.07 % at the protein level and demonstrated potent antitumor effects in HepG2 tumor-bearing humanized mice model with tumor sizes decreasing from ∼1300 mm^3^ to undetectable on day 25 post-treatment. This approach differs from conventional subcellular targeting by enabling exosomes to evade lysosomal trafficking, thereby reducing drug degradation by lysosomes, which also represents an application of subcellular targeting.

Potential applications of lysosome-targeted therapy extend far beyond current achievements (Li et al., 2020). In summary, current research on lysosome-targeted therapeutic strategies focuses on two major approaches. The first actively exploits the lysosomal microenvironment (e.g., pH responsiveness, enzymatic degradation) to achieve controlled drug release, while the second optimizes drug delivery by modulating the endosome-lysosome pathway. These strategies offer distinct advantages: the former maximizes the lysosome's role as a “drug-processing factory”, while the latter prevents premature degradation of therapeutic payloads. As a key organelle responsible for clearing foreign substances, the lysosome holds significant therapeutic potential for various cancer types, whether through evading its destructive effects or harnessing the enzymes it releases for targeted killing.

### 3.2 Nucleus

The nucleus serves as the central hub for critical nucleic acid-dependent cellular processes, such as proliferation and apoptosis. As such, it plays a pivotal role in tumorigenesis and progression. This central positioning grants nucleus-targeted cancer therapy unique advantages: direct nuclear disruption is lethal to tumor cells, while interfering with intranuclear processes may overcome drug resistance. However, conventional chemotherapeutic drugs face multiple biological hurdles—for instance, less than 5 % of administered cisplatin or doxorubicin reaches the nucleus (Cao et al., 2019; Lasorsa et al., 2019). And photothermal or photodynamic therapy also suffers from the limitation of short diffusion distances. Moreover, despite their broad tumor-targeting capability, translocation of exosomes into the tumor cell nuclei is inefficient, limiting therapeutic efficacy and contributing to severe systemic toxicity. Thus, developing highly efficient and precise nuclear-targeted exosome delivery systems is crucial to overcoming current barriers in treatment.

**Fig. 2:**
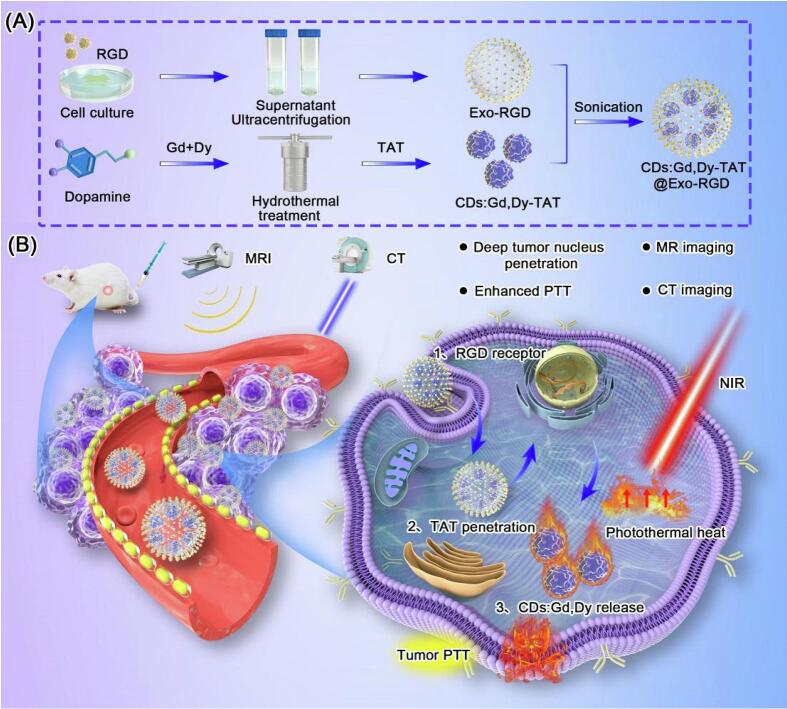
The Exo-RGD/CDs:Gd,Dy-TAT system combines nuclear targeting (TAT), tumor homing (RGD), and dual-modal imaging (MRI/CT) to enable near-infrared (NIR)-triggered photothermal destruction of tumor nuclei. Reprinted from (Yang et al., 2021); licensed under CC BY 4.0.

Engineered nuclear localization signal (NLS) peptides exhibit advantages in active targeting (Cao et al., 2019). The chimeric peptide-engineered exosome (ChIP-Exo) system integrates photodynamic therapy with nuclear targeting (Cheng et al., 2019). This system employs a spatiotemporally controlled two-stage therapeutic model utilizing a C16 alkyl chain to anchor the exosome membrane, an NLS peptide (PKKKRKV) for nuclear localization, and the photosensitizer Protoporphyrin IX (PpIX) to generate ROS. Initially, light irradiation triggers plasma membrane disruption to enhance cellular internalization, and subsequently, nuclear ROS directly damages DNA. This dual-stage nucleus-targeting strategy resulted in greater than 30 % improvement in the therapeutic effect as demonstrated by the reduced tumor weight (approx. 0.2 g) when compared to the single-stage treatment (0.3 g) (Female BALB/c mouse model bearing subcutaneous 4 T1 breast tumors). This sequential design not only improves tumor suppression rates but also significantly reduces systemic toxicity (the blood routine analysis). Moreover, it is precisely through achieving nuclear targeting during the second light irradiation that reactive oxygen species can exert their maximum cytotoxic effect within their limited diffusion range, which underscores the critical role of subcellular targeting in this design. Another example is the trans-activator of transcription (TAT) peptide integrated into a multifunctional exosome platform (CDs:Gd,Dy-TAT@Exo-RGD) (Zhang et al., 2020a). In this system, dopamine was carbonized via hydrothermal treatment to form a carbon core, which was then modified with gadolinium (Gd^3+^) and dysprosium (Dy^3+^) ions and conjugated with TAT. The resulting construct was further loaded into RGD peptide-modified macrophage-derived exosomes for targeted delivery to 4 T1 breast cancer and HeLa (cervical cancer) tumor models. The basis of this system is that the carbon core-mediated photothermal therapy would effectively kill tumor cells, as incorporation of the TAT peptide (avoiding energy dissipation during cytoplasmic transmission) enhanced in vitro (4 T1 and HeLa cells) tumor cell killing by ∼25 % and improved in vivo (BALB/c mouse syngeneic 4 T1 breast carcinoma transplantation model) targeting efficiency by about 30 % (Fig. 2) (Yang et al., 2021). Compared to NLS peptides, the TAT peptide exhibits unique membrane-penetrating ability, enabling nuclear entry without requiring transport proteins. Although its targeting specificity is slightly inferior to that of NLS peptides, TAT still demonstrates favorable nuclear accumulation efficacy for photothermal therapy.

In addition to active targeting, passive nuclear targeting can also be achieved by optimizing exosome physicochemical properties. Unmodified DOX-loaded exosome complexes rapidly translocated into the nucleus within 1 h (Pan et al., 2021), and this was primarily attributed to three key factors: (1) The positively charged amino-sugars on DOX enable electrostatic binding to the DNA phosphate backbone, (2) co-delivered Fe_3_O_4_ nanoparticles undergo the Fenton reaction to generate hydroxyl radicals which enhance nuclear membrane permeability, and (3) the exosomal carrier effectively controls drug-loaded particle size in line with a previous report that only nanoparticles smaller than 10 nm can efficiently traverse nuclear pore complexes (Huo et al., 2014).

In summary, the selection of nuclear targeting strategies should be tailored to therapeutic requirements: active targeting systems employing NLS peptides prove more suitable for precise strikes, whereas passive targeting may offer superior advantages when combined with therapies that intrinsically modify nuclear membrane permeability (e.g., photodynamic therapy) (Tu et al., 2020). Nevertheless, these strategies are not mutually exclusive—future research could explore the integration of active targeting peptides with physicochemical optimization. For instance, development of smart exosomes simultaneously equipped with modified NLS peptides and optimal particle size may establish a multimodal synergistic approach. Such advanced systems could significantly enhance nuclear delivery efficiency and overcome tumor drug resistance.

### 3.3 Mitochondria

Mitochondria, as the cellular energy hubs, are unique targets in cancer therapy and precision treatment. The mitochondrial membrane potential (ΔΨm) is 30–60 mV higher than other organelles, while tumor cells exhibit elevated ΔΨm compared to normal cells—this electrochemical gradient provides a natural advantage for charge-dependent targeting. In addition, the high dependence of tumor cells on mitochondrial energy metabolism renders them metabolically vulnerable; disrupting oxidative phosphorylation (OXPHOS) or inducing mitochondrial dysfunction can effectively kill cancer cells. However, conventional mitochondria-targeting drugs face challenges such as low delivery efficiency and off-target toxicity. Exosomes can be readily modified with high membrane-potential substances to target mitochondria while effectively delivering energy-disrupting drugs.

**Fig. 3:**
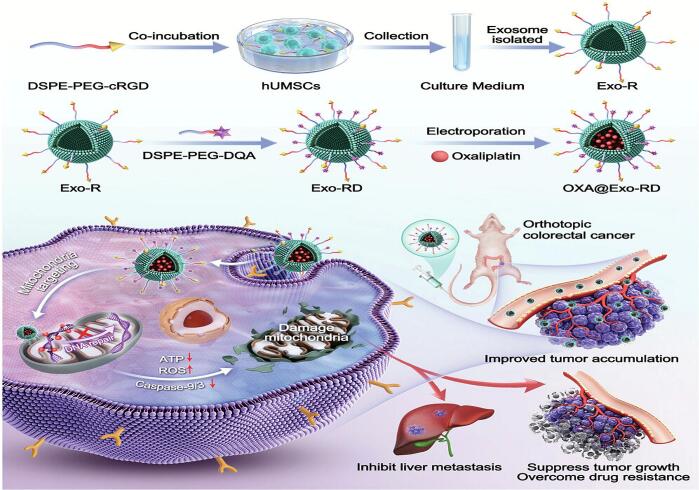
OXA@Exo-RD employs integrin/mitochondria-targeting exosomes to co-deliver oxaliplatin (OXA) to colorectal cancer (CRC) cells and mitochondria. OXA induces mitochondrial dysfunction to alleviate CRC cells' chemoresistance and enable the inhibition of metastasis. Reprinted from (Wang et al., 2025a); licensed under CC BY 4.0.

Current exosome-based mitochondrial targeting strategies primarily rely on charge-dependent mechanisms for targeting. Among charge-dependent strategies, the OXA@Exo-RD system conjugates mesenchymal stem cell-derived exosomes with cRGD peptide (targeting integrin ανβ3, cellular targeting) and dehydroabietic amine (DQA, a mitochondrial-targeting molecule) (Wang et al., 2025a). This system adopts a triple-targeting mechanism of exosome-mediated tumor accumulation, cRGD-enhanced internalization, and DQA mitochondrial localization (Fig. 3). As a DLC molecule, DQA selectively accumulates in mitochondria, dissipating MMP, inducing ROS, and inhibiting ATP synthesis to kill cancer cells. OXA acts by targeting poorly repairable mtDNA, reducing therapeutic failure risk. Together, they synergistically activate apoptosis, markedly upregulating Caspase-3, Caspase-9, and PARP expression. The incorporation of DQA enhanced mitochondrial targeting efficiency, as evidenced by the increase in Pearson's colocalization coefficient from 0.49 to 0.94 (in HCT116/OXA cells). This approach, when examined using in vivo experiments (HCT116/OXA subcutaneous xenograft model in BALB/c nude mice), led to a further reduction of over 30 % in both tumor volume and weight after three weeks of treatment. Similarly, the cationic property of triphenylphosphonium (TPP^+^) was leveraged to achieve mitochondrial co-delivery of the sonosensitizer Chlorin e6 (Ce6) and the glycolytic inhibitor FX11 (Nguyen Cao et al., 2023). TPP utilizes the mitochondrial membrane potential for targeted delivery. Upon ultrasound activation, the system generates ROS to directly cause mitochondrial oxidative damage, resulting in membrane potential collapse, structural disruption, and initiation of the mitochondrial apoptosis pathway. Meanwhile, FX11 inhibits the glycolytic enzyme LDHA, cutting off energy supply and further weakening the cell's capacity to repair oxidative damage. Ultimately, FX11 acts synergistically with T-Ce6-induced mitochondrial damage to significantly enhance lethality. The final formulation mediated a 90 % reduction in tumor burden (MCF-7 xenograft mouse model), with mitochondrial-targeting T-Ce6 conferring an additional 20 % improvement in targeting efficiency and a corresponding 30 % increase in overall treatment efficacy (MCF-7 human breast cancer cells). To date, other mitochondrial-targeting agents, including rhodamine 123 and mitochondrial targeting signal peptides (MTS), also exploit the electrochemical gradient for mitochondrial accumulation (Pathak et al., 2015; Dou et al., 2022). However, their application in exosome-based tumor mitochondrial targeting remains unexplored, presenting a promising direction for future research. However, exosome-mediated mitochondrial targeting still falls short of efficient lysosomal escape. As exosomes use the endocytic pathway, their transport to lysosomes compromises both targeting and payload delivery efficiency. The EXO-DVDMS system can overcome this limitation by exploiting the acidic lysosomal environment to trigger drug release, followed by ultrasound-induced lysosomal membrane disruption (Liu et al., 2019). This dual action approach allows positively charged DVDMS to escape into the cytoplasm and subsequently accumulate in mitochondria. This “lysosomal escape followed by mitochondrial targeting” cascade strategy effectively enhances intramitochondrial drug concentrations to significantly improve therapeutic outcomes. When using TPP mitochondrial-targeted exosomes loaded with doxorubicin (DOX) and carvedilol (CAR), (Soudi et al., 2025) found that the combination of the two drugs unexpectedly reduced apoptosis in breast cancer cells. The reason may be that CAR, by inhibiting mitochondrial respiratory chain complex I, decreased the generation of semiquinone intermediates produced by DOX redox cycling within mitochondria, thereby attenuating the burst of ROS. This result highlights the importance of rational design and mechanistic synergy in drug combination strategies for subcellular targeting.

Tumor cells hijacking immune cell mitochondria through tunneling nanotubes (TNTs) presents a new challenge (Hanna et al., 2017; Kidwell et al., 2023). Future studies could look into simultaneously targeting tumor cell mitochondria to induce apoptosis while disrupting TNTs formation (e.g., using farnesyltransferase inhibitors) to prevent metabolic compensation(Leng et al., 2025). This integrated approach addresses current limitations by combining tumor cell elimination, disruption of energy supplies, and prevention of mitochondrial theft (Vercellino and Sazanov, 2022; Li et al., 2021).

In summary, exosome-mediated mitochondrial-targeted therapy demonstrates significant advantages. Future directions should focus on the development of novel targeting ligands (e.g., MTS peptides), optimization of lysosomal escape efficiency and the design of comprehensive metabolic blockade strategies. These advancements are expected to facilitate crucial progress toward clinical translation of mitochondrial-targeted therapies.

### 3.4 Endoplasmic reticulum

The endoplasmic reticulum (ER) plays a dual critical role in tumorigenesis and progression. As the central hub for protein synthesis and folding, the aberrant proliferation of tumor cells leads to a significant increase in ER protein synthesis load. Conversely, activation of ER stress responses can directly influence tumor cell survival and metastasis (Fu et al., 2021). These biological characteristics position the ER as a highly promising therapeutic target. Exosomes offer an innovative solution for ER-targeted therapy by leveraging their natural internalization pathway through the ER-Golgi system (Liu et al., 2024).

**Fig. 4:**
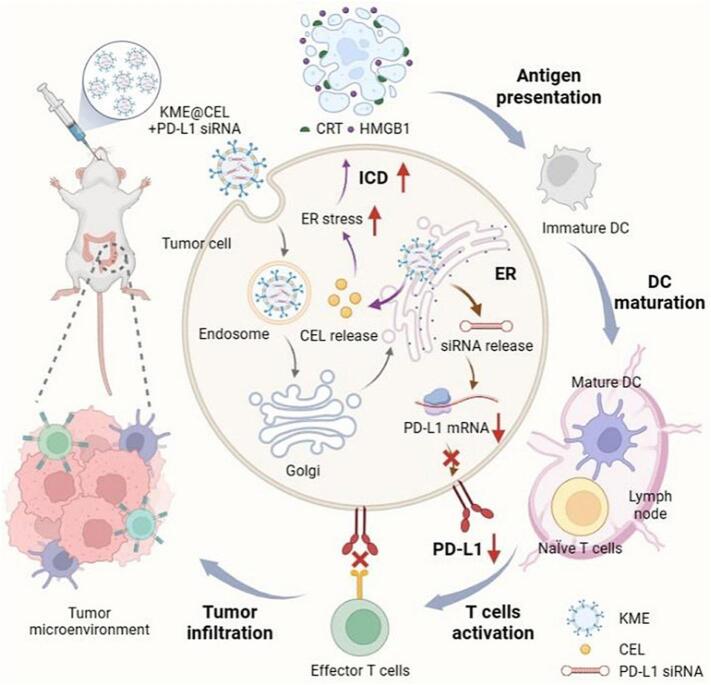
KDEL-modified milk exosomes (KME) deliver drugs to the endoplasmic reticulum (ER) via Golgi-ER trafficking. The CEL-PD-L1 siRNA complex boosts immunogenic cell death and, by blocking PD-L1 enhances anti-tumor immunity. Reprinted from(Wang et al., 2024); Copyright 2024, Elsevier.

Current strategies for exosome-mediated ER targeting can be classified into receptor-mediated active targeting and utilization of natural sorting pathways. The Lys-Asp-Glu-Leu (KDEL)-modified exosome system represents a prototypical example of receptor-mediated targeting. It is essential to recognize that PD-L1 is a key protein mediating tumor immune evasion, while siRNA can specifically bind to and degrade its corresponding mRNA. As the endoplasmic reticulum (ER) serves as the site for PD-L1 protein synthesis, its mRNA must be transported nearby to enable efficient translation by ribosomes. Therefore, designing siRNA targeted to the ER region can effectively degrade PD-L1 mRNA locally, thereby inhibiting protein expression and offering a novel strategy for immunotherapy. Building on this rationale, milk-derived exosomes were used as carriers and incorporated KDEL targeting peptides to achieve ER-specific delivery. This dual-loading system simultaneously carried the chemotherapeutic drug Celastrol (CEL) and PD-L1 (Programmed Death-Ligand 1) siRNA (Fig. 4). Following oral administration, the KDEL sequence directs exosome transport through the Golgi-ER pathway, significantly enhancing both CEL-induced immunogenic cell death and siRNA-mediated gene silencing effects. Furthermore, the ER-targeting KDEL sequence group reduced relative tumor bioluminescence intensity from a score of 4 to almost undetectable levels with similar outcomes visualized from survival data (CT26-Luc cell-based orthotopic colorectal cancer model in BALB/c mice) (Wang et al., 2024). Taken together, this design successfully achieved synergistic enhancement of chemotherapy and immunotherapy. Another approach utilized modulation of the physical morphology of exosomes (utilization of natural sorting pathways) for targeted delivery. It has been reported that rod-shaped exosomes selectively accumulate in the ER through caveolin-mediated endocytosis, while spherical exosomes are predominantly captured by lysosomes (Huang et al., 2021). When loaded with curcumin and modified with folic acid, the rod-shaped exosomes significantly enhanced ER stress-induced apoptosis in colorectal cancer models. All rod-shaped exosomes exhibited Pearson's colocalization coefficients of approximately 0.1 with lysosomes and 0.5 with the ER. In contrast, all spherical exosomes showed coefficients of approximately 0.6 with lysosomes and only 0.05 with the ER (HCT116 cells, A549 cells and HeLa cells) (Liang et al., 2024). Of note, the selective mechanisms through which exosomes of different shapes engage specific intracellular targeting pathways remain poorly understood and warrant further investigation. This finding highlight the critical influence of exosome physical characteristics on biological behavior, providing novel design principles for targeted delivery systems.

Taken together, both engineered targeting peptides and natural sorting mechanisms enable efficient drug enrichment in the ER while avoiding lysosomal degradation. These targeting strategies induce ER stress to directly kill tumor cells or disrupt protein synthesis to inhibit pro-tumor pathways, and also activate immunogenic cell death to enhance antitumor immune responses. The innovative approach of controlling exosome morphology has opened new possibilities for ER-targeted therapy, guiding further exploration of physical property-based targeting strategies. Notably, given the close functional and physiological relationship between the Golgi apparatus and ER, similar targeting strategies could be developed for Golgi-targeted cancer therapy.

The research summary on subcellular targeting is presented in Table 5.

**Table 5:** Exosome-based subcellular targeting and therapeutic materials and strategies toward tumors.

Source of exosomes	Tumor types	Materials for tumor cell targeting	Subcellular targeting strategy			Therapeutic strategy		References
			Subcellular targeting sites	Core materials	Key mechanisms	Core materials	Modality	
MSCs	TNBC	MF (Carboxylated Fe₃O₄)	Lysosome	**–**	Natural internalization pathways of exosomes	DOX	Chemotherapy	(Xu et al., 2024)
4 T1 cells	Breast	Ultrasound, naturally targeted	Lysosome	–	Natural internalization pathways of exosomes	Therapeutic ultrasound	Ultrasonic destruction	(Liu et al., 2019)
BMSCs cells	Hepatocellular Carcinoma	Naturally targeted, apoA1	Lysosome	apoA1	SR-B1 pathway	5′-cholesterol siRNA	Immunotherapy	(Zhang et al., 2024)
MCF10A cells, hFOB 1.19 cells	Breast	Naturally targeted	Late endosome	WGA	Non-lysosomal pathway	AgCu-NPs	Oxidative stress, cytotoxicity	(Ashraf et al., 2022)
Mouse blood	Breast, cervical, melanoma, colorectal	ChIP	Nucleus	NLS	NPC mediated transport	Photosensitizer (PpIX)	Dual-stage photodynamic therapy	(Cheng et al., 2019)
Raw 264.7 cells	Breast, cervical	RGD peptide	Nucleus	TAT peptide	NPC mediated transport	CDs	PTT	(Yang et al., 2021)
Urine from prostate cancer patients	Prostate	Naturally targeted	Nucleus	–	Particle size	Fe₃O₄, DOX	Chemodynamic therapy	(Pan et al., 2021)
4 T1 cells	Breast	Ultrasound, naturally targeted	Mitochondrion	DVDMS	Lipophilic	DVDMS	SDT	(Liu et al., 2019)
hUMSCs cells	CRC	cRGD peptide	Mitochondrion	DQA	ΔΨm	OXA, DQA	Chemotherapy, mitochondrial dysfunction	(Wang et al., 2025a)
HEK-293 T cells	Breast	Naturally targeted	Mitochondrion	TPP	ΔΨm	Sonosensitizer (Ce6), potent inhibitor of LDHA (FX11)	SDT, starvation therapy	(Nguyen Cao et al., 2023)
MCF-7 cells MDA-MB-231 cells	Breast	Naturally targeted	Mitochondrion	TPP	ΔΨm	DOX, CAR	Chemotherapy,	(Soudi et al., 2025)
CT26 cells	Colorectal	Naturally targeted	Mitochondrion	Mesoporous CaCO₃ NPs)	MCU	Mesoporous CaCO₃ NPs, Cur	Calcium overload therapy, SDT	(Li et al., 2022c)
Milk	Colorectal	–	ER	KDEL peptide	KDEL receptor	CEL, PD-L1 siRNA	Chemotherapy, immunotherapy	(Wang et al., 2024)
Milk, ginger, HepG2 cells	Colorectal	Folate	ER	Nanorods	Shape	Cur	ER-stress	(Liang et al., 2024)

Abbreviations list: TNBC, Triple-negative breast cancer; MF, Magnetic field; DOX, Doxorubicin; apoA1, Apolipoprotein A1; SR-B1, Scavenger receptor class B type 1; WGA, Wheat Germ Agglutinin; NPs, Nanoparticles; ChIP, Chimeric peptide; NLS, Nuclear localization signal; NPC, Nuclear pore complex; PpIX, Protoporphyrin IX; RGD, Arginine-Glycine-Aspartic acid; TAT, Trans-activator of transcription; CDs, Carbon dots; PTT, Photothermal therapy; DVDMS, Sinoporphyrin sodium; SDT, Sonodynamic therapy; CRC, Colorectal Cancer; QDA, Dequalinium; ΔΨm, Mitochondrial membrane potential; OXA, oxaliplatin; TPP, Triphenylphosphonium; CAR, Carvedilol; Ce6, Chlorin e6; LDHA, Lactate dehydrogenase A; MCU, Mitochondrial calcium uniporter; Cur, Curcumin; ER, Endoplasmic reticulum; CEL, Celastrol; PD-L1, Programmed death-ligand 1.

## 4 Conclusions and perspectives

Exosomes, as natural delivery vehicles, have tremendous potential in cancer therapy due to their superior biocompatibility, notable tissue penetration capability, and minimal side effects. Hence, exosomes represent an untapped therapeutic treasure trove. However, to fully unlock this potential requires strategic selection of exosome subtypes based on tumor-specific characteristics (including tumorigenesis, progression, metastasis and drug resistance patterns), coupled with precision engineering for target specificity and therapeutic mechanisms to achieve optimal treatment efficacy and safety (Severic et al., 2021; Deng et al., 2024). Nonetheless, clinical translation of exosome-based therapies still faces multiple challenges. Rapid clearance of exosomes by the liver and kidneys, limited targeting efficiency, and potential tumor resistance all constrain therapeutic applications (Li et al., 2025). To overcome these limitations, researchers are shifting their focus toward more precise targeting strategies, specifically directing therapeutic agents to tumor-specific subcellular structures. This subcellular-level precision targeting strategy offers two critical advantages. Precision targeting enables the penetration of traditional membrane barriers to substantially enhance drug delivery and efficacy, and also capitalizes on the relative autonomy of subcellular structures to delay or even eliminate drug resistance (Liu et al., 2020). Targeting subcellular structures represents a paradigm shift in overcoming current bottlenecks in exosome-based cancer therapy.

When designing and preparing subcellular-targeting exosome complexes, several critical considerations must be addressed. First, as nanoscale vesicles, exosomes have limited drug-loading capacity, necessitating optimal utilization of both their internal space and membrane structure (Caponnetto et al., 2017). During the loading process, strict control over drug molecule size is essential to ensure effective encapsulation. Stratified loading such that hydrophilic drugs are loaded into the aqueous lumen while hydrophobic compounds are embedded within the lipid bilayer, thereby maximizing loading efficiency, is a promising strategy (Bao et al., 2025). Secondly, the exosome intracellular trafficking pathway directly determines therapeutic outcomes (Arya et al., 2024; Pham et al., 2023). Exosomes may enter cells via multiple routes: the endosome-lysosome pathway, endosome-Golgi-ER route, or even direct membrane penetration. Precise modifications are therefore required based on the objectives of the therapy: exosome surface engineering can enhance tumor targeting (Belhadj et al., 2020), while incorporation of lysosomal escape elements (e.g., GALA peptides, a 30 amino acid synthetic peptide with a glu-ala-leu-ala (EALA) repeat that also contains a histidine and tryptophan residue) or subcellular structures localization signals (e.g., NLS, MTS) will ensure that drugs bypass endosomal/lysosomal barriers and reach the target subcellular structures (Chen et al., 2021). Thirdly, a thorough understanding of the characteristics of subcellular structures is paramount. Each subcellular structure possesses unique physicochemical and biological properties. For instance, as the cellular powerhouse and a site sensitive to ROS, mitochondria can be targeted via their high membrane potential and negative charge to induce energy depletion and trigger apoptotic pathways. And the nucleus, as the functional and regulatory center of the cell, is involved in initiating life activities and mediating drug resistance, making it a crucial therapeutic target. Meanwhile, the ER contains abundant KDEL receptors for ligand-directed delivery (Wang et al., 2025b), while the presence of nuclear peptides allows translocation across the nuclear envelope barrier (Du et al., 2023). These intrinsic properties provide the foundation for developing precision-targeted drug delivery systems.

On the clinical front, exosome innate biocompatibility, modifiable targeting capability, and substantial drug-loading capacity offer novel therapeutic strategies for diseases like cancer and inflammatory conditions. Moreover, subcellular-targeted exosomes further highlight their distinct advantages on this basis: in terms of delivery efficiency, reverse utilization of subcellular targeting to evade lysosomal clearance reduces drug loss; in drug protection, they enable precise delivery of degradable nucleic acids to sites of action such as the endoplasmic reticulum, preventing inactivation; in local therapies (e.g., photothermal therapy), they allow the enrichment of therapeutic agents within target organelles, thereby maximizing efficacy. Furthermore, this strategy can reduce dosing-related toxicity by enhancing targeting precision, and by precisely delivering drugs to targets such as the nucleus, it inhibits DNA repair to delay drug resistance. Significant limitations exist that prevent translational application of both conventional exosome drug carriers and exosomes designed for subcellular targeting. Firstly, exosome-targeting efficiency remains a challenge. In the highly complex internal environment of the body, specificity of engineered exosomes may be compromised by non-specific interactions (Araldi et al., 2023). A key example is the exosome off-target binding to inflammatory cells that also express PD-1, while the innate homing tendency of exosomes further exacerbates non-specific accumulation (De Miguel and Calvo, 2020). In recent years, the application of computers and artificial intelligence in biological screening has matured, demonstrating significant potential. AI technologies, represented by drug screening and protein structure prediction, hold promise for providing efficient and precise new pathways for screening and designing peptide ligands for subcellular-targeted exosomes(Alnasraui et al., 2024; Hussein et al., 2024).

Secondly, the safety profile of exosomes has not been fully dissected. As exosomes carry complex cargo such as nucleic acids and proteins, validating their safety requires long-term data (Welsh et al., 2024). Concomitantly, it remains unclear whether the materials used during exosome engineering would alter their inherent low immunogenicity, in addition to the additional risks posed by their dual role in promoting and suppressing tumors (He et al., 2022b). During the transition from basic research to clinical translation, 3D tumor spheroids or organoid models can address the limitations of traditional validation methods and help tackle safety challenges posed by engineered exosomes(Tchoryk et al., 2019). Organoids are derived from progenitor or differentiated cells of human tissues, replicating the complex structure and function of the corresponding tissue, thereby more accurately simulating drug delivery barriers and aiding in efficacy evaluation(Zhao et al., 2022). 3D tumor spheroids are rapidly constructed (typically within days to a week), suitable for high-throughput screening and better replicate the tissue-penetrating ability of exosomes. However, they may inadequately mimic the tumor microenvironment and offer limited support for immunotherapy research(He et al., 2022a).

Finally, exosome-based carriers must overcome numerous obstacles that prevent translating preclinical research findings into the clinical setting. In terms of production and manufacturing, low yield and high costs present major hurdles. Specifically, natural exosome secretion is relatively inefficient, and significant challenges exist when attempting large-scale isolation, characterization, and purification of exosomes(Batista et al., 2024). Moreover, exosomes are classified as Advanced Therapy Medicinal Products (ATMPs) and require strict Good Manufacturing Practice (GMP) compliance (Chen et al., 2020). Among these, ensuring the consistency, comparability, and quality controllability of engineered exosome products is the fundamental basis for achieving their clinical translation and large-scale application. There is an urgent need to establish high-throughput, automated exosome engineering platforms and to develop standardized, systematic characterization and quality control systems, thereby effectively controlling and reducing batch-to-batch variability at the source of the manufacturing process. As proposed by (Lu et al., 2024), this dual-mode detection technology offers a highly sensitive method for detecting tumor-derived exosomes through a multifunctional peptide-graphene sensing platform, laying the groundwork for clinical translation.

For subcellular-targeted exosome strategies, their effectiveness primarily depends on whether the drug has a clear subcellular organelle target. This approach is suitable for drugs that need to be delivered to specific organelles (such as mitochondria or the nucleus), such as siRNA, while offering limited benefits for drugs widely distributed in the cytoplasm, such as paclitaxel (Liu et al., 2020). Second, subcellular targeting lacks tumor specificity and may affect normal cells to the same extent (Zhen et al., 2021). If the cellular targeting efficiency of exosomes is insufficient, even a small amount of drugs delivered off-target to normal cells could exacerbate damage due to precise subcellular localization, such as cumulative mitochondrial toxicity or excessive immune responses (Soudi et al., 2025). AI-assisted targeting peptide design holds promise for improving specificity in the future. Furthermore, insufficient endosomal/lysosomal escape efficiency remains a common challenge for this strategy. Future efforts should integrate efficient escape mechanisms with subcellular targeting designs to synergistically enhance the overall efficiency of drug delivery to target subcellular structures.

With ongoing advanced research, subcellular-targeting exosome therapy is poised to overcome current limitations in exosome-based drug delivery systems. By optimizing loading strategies, precisely controlling internalization pathways, and developing subcellular-targeting exosome complexes, this novel therapeutic approach can significantly enhance treatment efficacy while minimizing systemic toxicity. This precision is further amplified by emerging technologies: bio-orthogonal labeling confers spatiotemporally precise organelle targeting, membrane hybridization optimizes the intracellular delivery kinetics of the carriers, and microneedle-assisted delivery addresses key bottlenecks in clinical translation(Zhang et al., 2023; Ragab, 2025). Such a breakthrough promises to revolutionize cancer therapy and direct the future of precision medicine.

## Declaration of generative AI in scientific writing

During the preparation of this work the authors used Deepseek in order to obtain suggestions for language modifications. After using this tool, the authors reviewed and edited the content as needed and take full responsibility for the content of the publication.

## CRediT authorship contribution statement

**Fengbo Liu:** Writing – original draft, Investigation, Formal analysis, Conceptualization. **Chengxing Xia:** Writing – review & editing, Visualization. **Hang Yu:** Project administration, Data curation. **Xiaofang Yang:** Formal analysis. **Liping Ge:** Data curation. **Chunwei Ye:** Writing – review & editing, Funding acquisition, Conceptualization.

## Funding sources

This work was supported by the Yunnan Revitalization Talent Support Program [Grant numbers XDYC-QNRC-2023-0184].

## Declaration of competing interest

The authors declare the following financial interests/personal relationships which may be considered as potential competing interests:

Chunwei Ye reports financial support was provided by the Yunnan Revitalization Talent Support Program. If there are other authors, they declare that they have no known competing financial interests or personal relationships that could have appeared to influence the work reported in this paper.

## Data Availability

Data sharing not applicable to this article as no datasets were generated or analysed during the current study.
